# Acute intoxication with diisopropylfluorophosphate promotes cellular senescence in the adult male rat brain

**DOI:** 10.3389/ftox.2024.1360359

**Published:** 2024-04-30

**Authors:** Yi-Hua Tsai, Eduardo A. González, Ana C. G. Grodzki, Donald A. Bruun, Naomi H. Saito, Danielle J. Harvey, Pamela J. Lein

**Affiliations:** ^1^ Department of Molecular Biosciences, School of Veterinary Medicine, University of California, Davis, Davis, CA, United States; ^2^ Department of Public Health Sciences, School of Medicine, University of California, Davis, Davis, CA, United States

**Keywords:** organophosphate, neuronal cell aging, neurodegeneration, p16, senescence-associated beta-galactosidase, seizure model

## Abstract

Acute intoxication with high levels of organophosphate (OP) cholinesterase inhibitors can cause cholinergic crisis, which is associated with acute, life-threatening parasympathomimetic symptoms, respiratory depression and seizures that can rapidly progress to status epilepticus (SE). Clinical and experimental data demonstrate that individuals who survive these acute neurotoxic effects often develop significant chronic morbidity, including behavioral deficits. The pathogenic mechanism(s) that link acute OP intoxication to chronic neurological deficits remain speculative. Cellular senescence has been linked to behavioral deficits associated with aging and neurodegenerative disease, but whether acute OP intoxication triggers cellular senescence in the brain has not been investigated. Here, we test this hypothesis in a rat model of acute intoxication with the OP diisopropylfluorophosphate (DFP). Adult male Sprague-Dawley rats were administered DFP (4 mg/kg, s.c.). Control animals were administered an equal volume (300 µL) of sterile phosphate-buffered saline (s.c.). Both groups were subsequently injected with atropine sulfate (2 mg/kg, i.m.) and 2-pralidoxime (25 mg/kg, i.m.). DFP triggered seizure activity within minutes that rapidly progressed to SE, as determined using behavioral seizure criteria. Brains were collected from animals at 1, 3, and 6 months post-exposure for immunohistochemical analyses of p16, a biomarker of cellular senescence. While there was no immunohistochemical evidence of cellular senescence at 1-month post-exposure, at 3- and 6-months post-exposure, p16 immunoreactivity was significantly increased in the CA3 and dentate gyrus of the hippocampus, amygdala, piriform cortex and thalamus, but not the CA1 region of the hippocampus or the somatosensory cortex. Co-localization of p16 immunoreactivity with cell-specific biomarkers, specifically, NeuN, GFAP, S100β, IBA1 and CD31, revealed that p16 expression in the brain of DFP animals is neuron-specific. The spatial distribution of p16-immunopositive cells overlapped with expression of senescence associated β-galactosidase and with degenerating neurons identified by FluoroJade-C (FJC) staining. The co-occurrence of p16 and FJC was positively correlated. This study implicates cellular senescence as a novel pathogenic mechanism underlying the chronic neurological deficits observed in individuals who survive OP-induced cholinergic crisis.

## 1 Introduction

Organophosphate cholinesterase inhibitors (OPs) are a family of potent neurotoxic chemicals that includes nerve agents and pesticides. OP poisoning from accidental or intentional (suicidal) exposures, war, or terrorism is a significant global public health issue (Eddleston and Phillips, 2004; Vogel, 2013; UN, 2017; Haley, 2018). It is estimated that acute OP intoxication annually causes 3 million life-threatening poisoning cases and 250,000 deaths worldwide (Pereira et al., 2014; Mew et al., 2017).

The canonical mechanism of acute OP neurotoxicity is acetylcholinesterase inhibition, which results in hyperstimulation of muscarinic and nicotinic cholinergic receptors in the peripheral and central nervous systems due to excessive accumulation of acetylcholine in the synapse (Pope et al., 2005). Acute inhibition of acetylcholinesterase by ≥ 60%–70% triggers cholinergic crisis, a clinical toxidrome that includes seizures that rapidly progress to *status epilepticus* (SE) (Pereira et al., 2014; Richardson et al., 2019). While current medical countermeasures for OPs are effective in reducing mortality, unless administered within minutes following exposure, they do not effectively terminate OP-induced seizures or protect against delayed brain damage and long-term morbidity (Eddleston et al., 2008; Chen, 2012; Jett and Spriggs, 2020). Clinical and experimental reports describe long-term neurologic effects, such as neuropsychiatric and cognitive deficits, in those who survive acute OP intoxication (Chen, 2012, reviewed in Figueiredo et al., 2018, reviewed in Jett et al., 2020). These observations coupled with the sobering fact that acutely intoxicating OP exposures remain significant public health threats, underscore the importance of better understanding the pathogenic mechanisms underlying adverse neurological outcomes so that more effective neuroprotective therapies can be found.

Impaired cognitive function has been reported in humans who survived acute OP exposures (Miyaki et al., 2005; Yamasue et al., 2007; Loh et al., 2010, reviewed in Jett et al., 2020, reviewed in Figueiredo et al., 2018, reviewed in Vale and Lotti, 2015). Survivors of the Tokyo subway sarin attack in 1995 have been reported to have not only deficits in cognitive function (Miyaki et al., 2005), but also structural brain damage and decreased gray matter volume in some brain regions (Yamasue et al., 2007). Data from preclinical models of acute OP intoxication (de Araujo Furtado et al., 2012; Deshpande et al., 2014; Pereira et al., 2014; Flannery et al., 2016; Guignet et al., 2020) corroborate human deficits in cognitive function. For instance, adult rats acutely intoxicated with the OP, diisopropylfluorophosphate (DFP) were observed to have impaired memory at 1-month post-exposure as assessed using the Morris water maze (Brewer et al., 2013), at 2 months post-exposure as evaluated by Pavlovian fear conditioning (Guignet et al., 2020), and at 3 months post-exposure using novel object recognition (Rojas et al., 2016).

Extensive experimental and epidemiological data implicate non-cholinergic mechanisms in a diverse range of neurotoxic outcomes associated with OPs (reviewed in Naughton and Terry, 2018; Tsai and Lein, 2021). In the context of the chronic neurotoxicity associated with acute OP intoxication, several non-cholinergic mechanisms are widely posited, including neuroinflammation (reviewed in Guignet and Lein, 2019, reviewed in Andrew and Lein, 2021) and oxidative stress (reviewed in Vanova et al., 2018, reviewed in Pearson and Patel, 2016). The spatiotemporal patterns of neuroinflammation and oxidative stress have recently been characterized in the rat model of acute DFP intoxication (Sisó et al., 2017; Putra et al., 2020a; Guignet et al., 2020; Rojas et al., 2022). However, few studies to date have determined whether experimental manipulation of neuroinflammation or oxidative stress ameliorate long-term effects of acute OP intoxication (Putra et al., 2020a; Putra et al., 2020b; Rojas et al., 2020).

Cellular senescence has been linked to cognitive impairment associated with neurodegenerative disease and age-related cognitive impairment (reviewed in Baker and Petersen, 2018, reviewed in Kritsilis et al., 2018). Cellular senescence, which was originally described as the finite capability of cell replication before experiencing stable growth arrest (Hayflick and Moorhead, 1961), is now thought to be a stress response triggered by a variety of intrinsic and extrinsic insults, such as oncogenic activation, oxidative and genotoxic stress, or mitochondrial dysfunction (reviewed in Kuilman et al., 2010, reviewed in McHugh and Gil, 2017). Cellular senescence can be beneficial or deleterious (reviewed in Burton and Krizhanovsky, 2014). For example, induction of cellular senescence can act to irreversibly halt proliferation of cells at risk for malignant transformation (reviewed in Campisi, 2013); conversely, persistent cellular senescence in aged tissues is thought to contribute to age-related pathologies (Baker et al., 2016, reviewed in van Deursen, 2014). Distinguishing characteristics of senescent cells include upregulated gene and protein expression of p16 and p21 (which are cyclin-dependent kinase inhibitors), chromatin reorganization, loss of nuclear lamin B1, adoption of a pro-inflammatory phenotype known as the senescence-associated secretory phenotype (SASP), and increased β-galactosidase (SA-βgal) activity (reviewed in Baker and Petersen, 2018, reviewed in Childs et al., 2017). While the role of cellular senescence in brain aging and neurodegenerative disease has yet to be fully elucidated, various cell types of the central nervous system have been found to display characteristics of senescence in the context of Alzheimer’s and Parkinson’s disease, including neurons, glial cells and endothelial cells (Arendt et al., 1998; Bhat et al., 2012; Chinta et al., 2018; Musi et al., 2018; Riessland et al., 2019; Graves and Baker, 2020).

Reports that repeated exposure to paraquat in a preclinical model of Parkinson’s disease promotes senescence of astrocytes (Chinta et al., 2018) were among the first evidence that exposure to neurotoxic chemicals can promote cellular senescence in the brain. Here, we leverage a well-characterized rat model of acute DFP intoxication to test the hypothesis that acute OP intoxication promotes cellular senescence in the brain. Cellular senescence was assessed using quantitative immunohistochemistry of biomarkers of cellular senescence during the 6 months following DFP-induced SE.

## 2 Materials and methods

### 2.1 Animals

Animals were maintained in facilities fully accredited by AAALAC International, and all studies were performed under protocols approved by the University of California, Davis Institutional Animal Care and Use Committee (IACUC protocol number 20165) in accordance with the ARRIVE guidelines and the National Institutes of Health Guide for the Care and Use of Laboratory Animals. Adult male Sprague-Dawley rats (8 ± 1 weeks old, 225–250 g) were purchased from Charles River Laboratories (Hollister, CA, United States), and upon receipt at the UC Davis vivarium were group housed 3 per cage in standard plastic shoebox cages with autoclaved corncob bedding. Animals were maintained in controlled environmental conditions (22°C ± 2°C, 40%–50% humidity, 12 h light-dark cycle) with *ad libitum* access to food and water. Animals were allowed to acclimate for at least 7 days before being used for experimentation.

### 2.2 DFP exposure paradigm and behavioral seizure monitoring

DFP was purchased from Sigma-Aldrich (St. Louis, MO, United States), and stocks were confirmed to have a purity of 90% ± 7% as determined by ^1^H-, ^13^C, ^19^F and ^31^P NMR methods (Gao et al., 2016). DFP aliquots were stored at −80°C to ensure chemical stability for over a year (Heiss et al., 2016). On the day of dosing, DFP was prepared in ice-cold sterile phosphate-buffered saline (PBS, 3.6 mM Na_2_HPO_4_, 1.4 mM NaH_2_PO_4_, 150 mM NaCl; pH 7.2) and administered at 4 mg/kg (s.c.) in the scruff of the neck. Control animals were administered a comparable volume (300 µL) of ice-cold sterile PBS. Rats (*n* = 4–6 per group) were randomly divided into DFP or vehicle control (VEH) groups using a random number generator. Both DFP and VEH groups received a combined intramuscular (i.m.) injection of atropine sulfate (2 mg/kg, purity ≥97%, Sigma-Aldrich) and 2-pralidoxime (2-PAM, 25 mg/kg, purity ≥99%, Sigma-Aldrich) in sterile saline (0.9% NaCl) in the inner thigh of the hind leg 1 min following DFP/VEH administration. This post-exposure treatment has been shown to significantly increase survival by reducing peripheral cholinergic symptoms associated with acute OP intoxication (Pessah et al., 2016).

**FIGURE 1:**
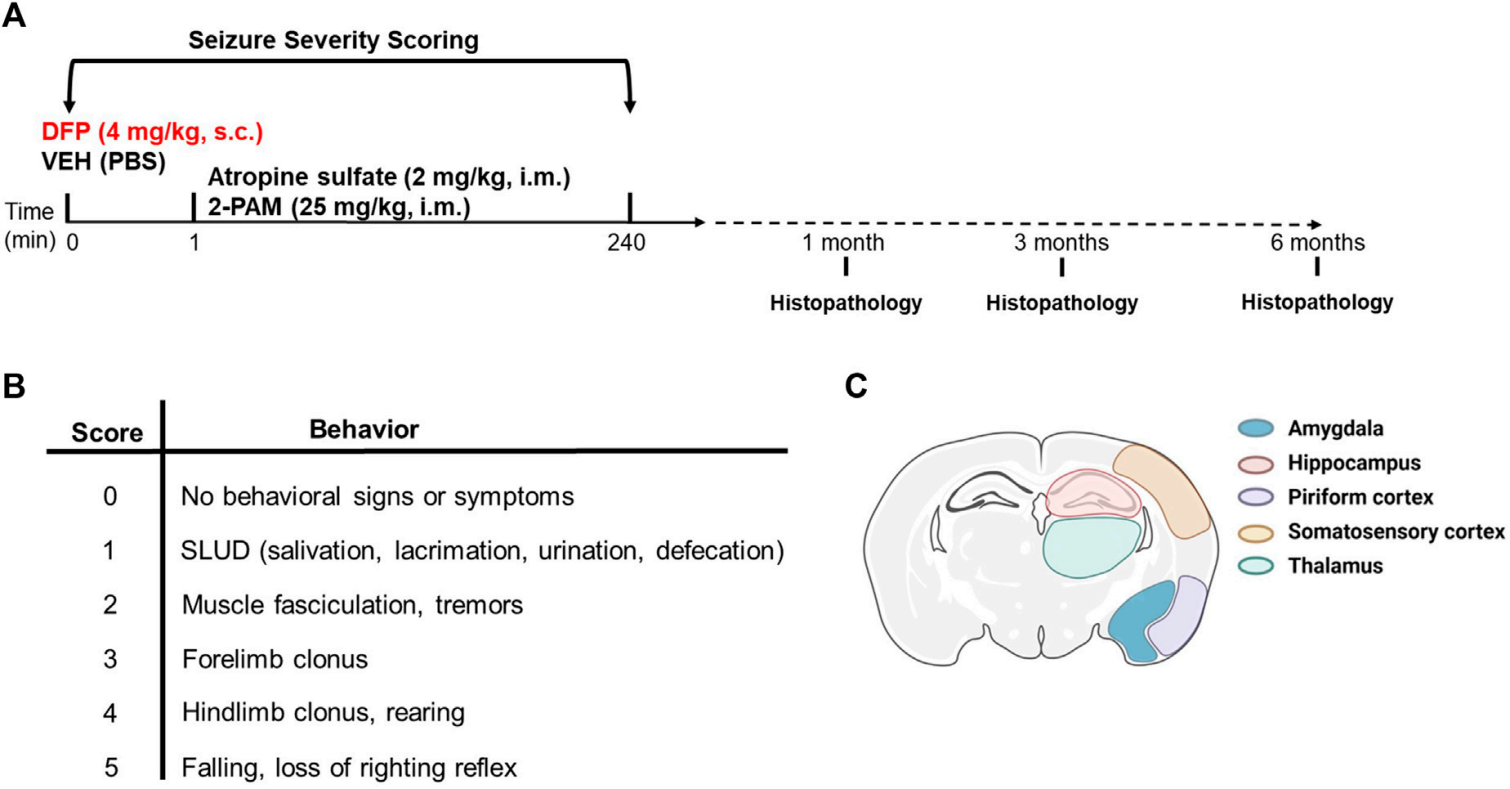
Experimental design. **(A)** Adult male Sprague-Dawley rats were treated with DFP (4 mg/kg, s.c.) or equivalent volume of vehicle (300 μL of PBS) at time 0. Both groups were administered atropine sulfate (2 mg/kg, i. m.) and 2-PAM (25 mg/kg, i. m.) 1 min later. Animals were euthanized at 1, 3, or 6 months post-DFP intoxication to collect tissues for immunohistochemistry. **(B)** Modified Racine scale used to score seizure behavior during the first 4 h post-DFP intoxication. **(C)** Illustration of the brain regions examined for neuropathology. Created with BioRender.com.

All animals were continuously monitored for seizure behavior for 4 h post-exposure. Seizure behavior was scored every 5 min for the first 2 h and then every 20 min for the next 2 h using a modified Racine scale as previously described (Deshpande et al., 2010). Briefly, seizure behavior scores ranged from 0 (no signs of symptoms) to 5 (rearing and falling, and loss of righting reflex) (see Figure 1). DFP-intoxicated animals with consecutive seizure scores ≥ 3 have previously been shown to be in SE as determined using electroencephalographic criteria (Deshpande et al., 2010). The DFP rats included in this study had consecutive seizure scores of ≥ 3 during the first 40 min after DFP injection. At the end of the 4 h seizure monitoring period, DFP animals were administered 10 mL of 5% dextrose in 0.9% isotonic saline (Baxter International, Deerfield, IL, United States) to replace fluids lost before being returned to their home cages. Soft chow was provided 3–5 days following DFP intoxication until the animals resumed normal consumption of water and solid food.

### 2.3 Histochemistry and immunohistochemistry

At 1, 3, and 6 months post-exposure, animals were humanely euthanized with 4% isoflurane in medical grade oxygen and subsequently perfused transcardially with PBS at a flow rate of 15 mL/min using a Masterflex peristaltic pump (Cole Parmer, Vernon Hills, IL, United States). Following euthanasia, brains were quickly excised from the skull and placed on ice. Each brain was bisected and the right hemisphere sliced into 2-mm thick coronal sections that were post-fixed in 4% (w/v) paraformaldehyde (PFA; Sigma-Aldrich) in phosphate buffer (0.1 M Na_2_HPO_4_, 0.1 M NaH_2_PO_4_, pH = 7.2) at 4°C overnight. Sections were then switched to 30% (w/v) sucrose (Sigma-Aldrich) in PBS for storage at 4°C until embedded and frozen in Tissue-Plus™ O.C.T. (Thermo Fisher Scientific, Waltham, MA, United States). Brain tissue blocks were cryosectioned into 10-μm thick coronal sections and stored at −80°C.

To detect degenerating neurons, FluoroJade-C (FJC, AG325, Millipore, Burlington, MA, United States) labeling was performed according to the manufacturer’s protocol. Briefly, after drying at 50°C for 10–15 min, sections were rinsed with 70% (v/v) ethanol for 2 min followed by a distilled water wash. Sections were incubated in 0.03% (w/v) potassium permanganate (KMnO_4_; Sigma-Aldrich) in distilled water for 10 min on a shaker table followed by a 2 min distilled water wash. Next, sections were incubated in a freshly prepared solution of 0.00015% FJC in 0.1% (v/v) acetic acid (Acros Organics, Geel, Belgium) in distilled water containing 0.5 μg/mL DAPI (Invitrogen, Carlsbad, CA, United States) for 10 min. Sections were then washed with distilled water, and dried at 50°C. Dried slides were cleared by immersion in chemical grade xylene (Thermo-Fisher Scientific) for 1 min before being coverslipped in Permount mounting medium (Thermo-Fisher Scientific).

For immunostaining, slides were air dried at room temperature before being rinsed with PBS. Antigen retrieval was performed by incubating the slides in 10 mM sodium citrate buffer (pH = 6.0) for 30 min in a rice cooker (Black & Decker, HS 2000). Following antigen retrieval, sections were washed 3 times with PBS then blocked for 1.5 h at room temperature in 5% (w/v) bovine serum albumin (Sigma-Aldrich) and 10% (v/v) normal goat serum (Vector Laboratories, Burlingame, CA, United States) in PBS containing 0.03% (v/v) Triton X-100 (Thermo-Fisher Scientific). Sections were then incubated with primary antibodies in blocking buffer overnight at 4°C. Primary antibodies used to assess neuropathology included mouse anti p16 (1:500, MA5-17142, RRID AB_2538613, Thermo-Fisher Scientific), rabbit anti-IBA1 (1:1000, 019-19741, RRID AB_839504, Wako Laboratory Chemicals, Richmond, VA, United States), guinea pig anti-GFAP (1:500, 173,004, RRID AB_10641162, Synaptic Systems, Göttingen, Germany), rabbit anti-S100β (1:300, ab52642, RRID AB_882426, Abcam, Cambridge, United Kindom), rabbit anti-NeuN (1:500, PA5-37407, RRID AB_2554049, Thermo Fisher Scientific), rabbit anti-CD31/PECAM-1 (1:250, NB100-2284, RRID AB_10002513, Novus Biologicals, Littleton, CO, United States).

The next day, sections were washed 3 times in PBS with 0.03% (v/v) Triton X-100 before incubation with secondary antibodies in blocking buffer for 1 h at room temperature. The following secondary antibodies were used: for anti-p16, goat anti-mouse IgG1 conjugated to Alexa Fluor 488 (1:500, A21121, RRID AB_2535764, Life Technologies, Carlsbad, CA, United States) or goat anti-mouse IgG1 conjugated to Alexa Fluor 568 (1:500, A21124, RRID AB_2535766, Life Technologies); for anti-IBA1, goat-anti rabbit IgG conjugated to Alexa Fluor 568 (1:1000, A11036, RRID AB_10563566, Life Technologies); for anti-GFAP, goat anti-guinea pig IgG conjugated to Alexa Fluor 647 (1:1000, A21450, RRID AB_2735091, Thermo-Fisher Scientific); for anti-NeuN, goat anti-rabbit IgG (H + L) conjugated to Alexa Fluor 488 (1:500, A11034, RRID AB_2576217, Life Technologies); and for anti-CD31, goat anti-rabbit IgG (H + L) conjugated to Alexa Fluor 647 (1:1000, A21245, RRID AB_2535813, Life Technologies). Negative controls, sections reacted with secondary antibodies only, were run with each batch. All slides were mounted in ProLong™ Gold Antifade with DAPI (Invitrogen, Waltham, MA, United States).

Sections were processed and stained for senescence associated β-galactosidase (SA-β-gal) using the CellEvent Senescence Green Detection Kit (ThermoFisher Scientific). For dual immunostaining and FJC labeling, sections were first processed for immunohistochemistry as described above, and then processed for FJC labeling with the following modifications of the manufacturer’s protocol: sections were immersed in 0.015% KMnO_4_ for 1 min followed by incubation in 0.0001% FJC solution for 10 min. The sections were rinsed with distilled water and coverslipped in Permount mounting medium.

### 2.4 High content imaging and image analysis

Fluorescent images were acquired using the high-content ImageXpress XL imaging system (Molecular Devices, Sunnyvale, CA, United States). Images of p16 immunoreactivity and FJC labeling in the following brain regions were acquired from two serial sections from each animal (*n* = 4–5 animals for VEH groups; *n* = 5–7 animals for DFP groups): amygdala, hippocampus (CA1, CA3, dentate gyrus), piriform and somatosensory cortex, and dorsolateral thalamus. The final image of each brain region for analysis was built by stitching multiple overlapping tiles that encompassed an entire brain region (MetaXpress Version 6.2.3.733). ImageJ imaging software (version 1.51n, NIH, United States) was applied to set the threshold for fluorescence intensity. Cell size and circularity were used to determine the number of FJC-positive cells per unit area (mm^2^). p16/NeuN immunoreactivity was assessed using a custom-designed module (MetaXpress Custom Module Editor Version 6.2.3.733) with respect to the percentage of NeuN-immunopositive cells (identified by DAPI staining) with colocalized p16 immunoreactivity. The custom-designed module is described in the Supplementary Material.

### 2.5 Statistics

For the histological analysis of neuronal expression of the senescence marker p16 and FJC-positive cell count, mixed-effects models (including animal-specific random effects) were fit to assess differences between exposure groups. Primary factors of interest included exposure (DFP, VEH), region (amygdala, CA3, CA1 and hilus of the hippocampus, somatosensory and piriform cortices, and thalamus), and time post-exposure (1, 3, and 6 months post-exposure). Interactions between the factors (exposure, region, and time point) were considered and the best model was chosen using Akaike Information Criterion. To better meet the assumptions of the model, the outcome was transformed using the natural logarithm after shifting all values by 1 to enable analyses of animals with no colocalization or no detectable FJC. Contrasts for group differences by time point and region (p16/NeuN colocalization) and exposure groups (FJC-positive cell count) were constructed and tested using a Wald test. Further contrasts were constructed between time points by region within each exposure group to assess the temporal differences in p16/NeuN colocalization. The Benjamini-Hochberg false discovery rate (FDR) was used to account for multiple comparisons across contrasts. Results are presented as geometric mean ratios (GMR) between exposure groups or between time points. Point estimates of the ratios and 95% confidence intervals are presented in the figures. When the confidence interval for the GMR includes 1, there is no statistical evidence of a difference between groups; similarly, when the confidence interval for the differences includes 0, there is no statistical evidence of a difference between groups. All analyses were performed using SAS software, version 9.4 with alpha set at 0.05. All reported significant results remained significant after the FDR procedure. Correlations between the number of cells expressing p16 and FJC-positive degenerating neurons were analyzed by Pearson’s correlation coefficient using Prism 9. 1. 2 (GraphPad Software).

## 3 Results

### 3.1 Acute DFP intoxication increased p16 immunoreactivity at 3-months post-exposure

**FIGURE 2:**
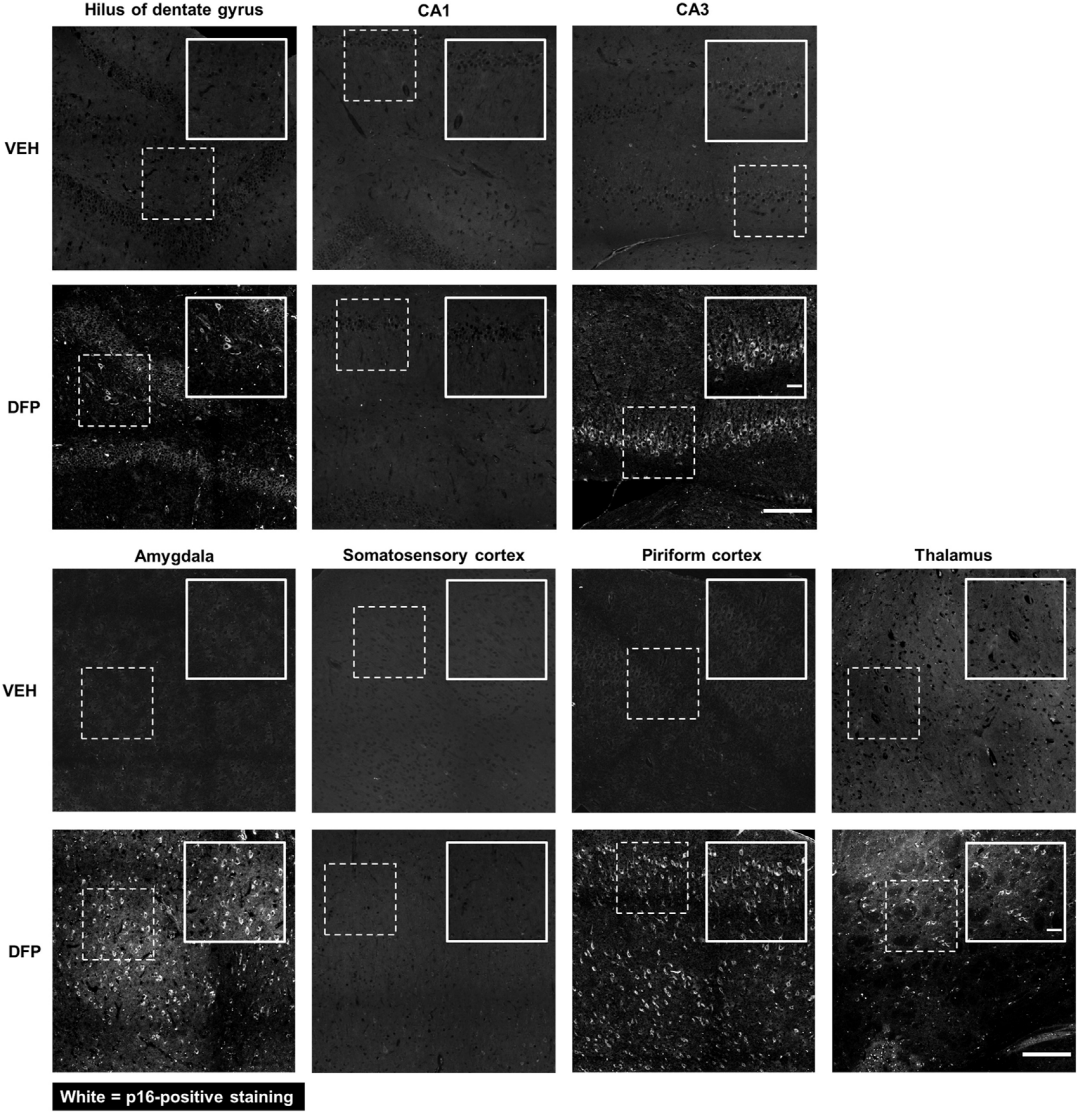
p16 Immunoreactivity was detected in specific brain regions at 3-months post-DFP intoxication. Representative photomicrographs showing p16-immunopositive cells in the hilus of the dentate gyrus, CA3 of the hippocampus, amygdala, piriform cortex, and thalamus. p16 Immunoreactivity was not detected in the CA1 of the hippocampus and the somatosensory cortex at 3-months post-intoxication. Dashed boxes identify the field in the lower magnification image (scale bar = 200 µm) that is shown at higher magnification in the inset box delineated by solid lines (scale bar = 50 µm).

p16 is a well-established biomarker of cellular senescence that is associated with aging-related pathologies (Baker et al., 2011). Relative to VEH controls, p16 expression was significantly increased in multiple brain regions in the DFP animals at 3-months post-exposure (Figure 2). In DFP animals, p16 immunopositive cells were detected in the hilus and CA3 subregions of the hippocampus, amygdala, piriform cortex, and thalamus. In contrast, there was negligible p16 expression in the CA1 of the hippocampus and somatosensory cortex of DFP animals.

### 3.2 p16 is expressed by neurons in the acute DFP rat model

**FIGURE 3:**
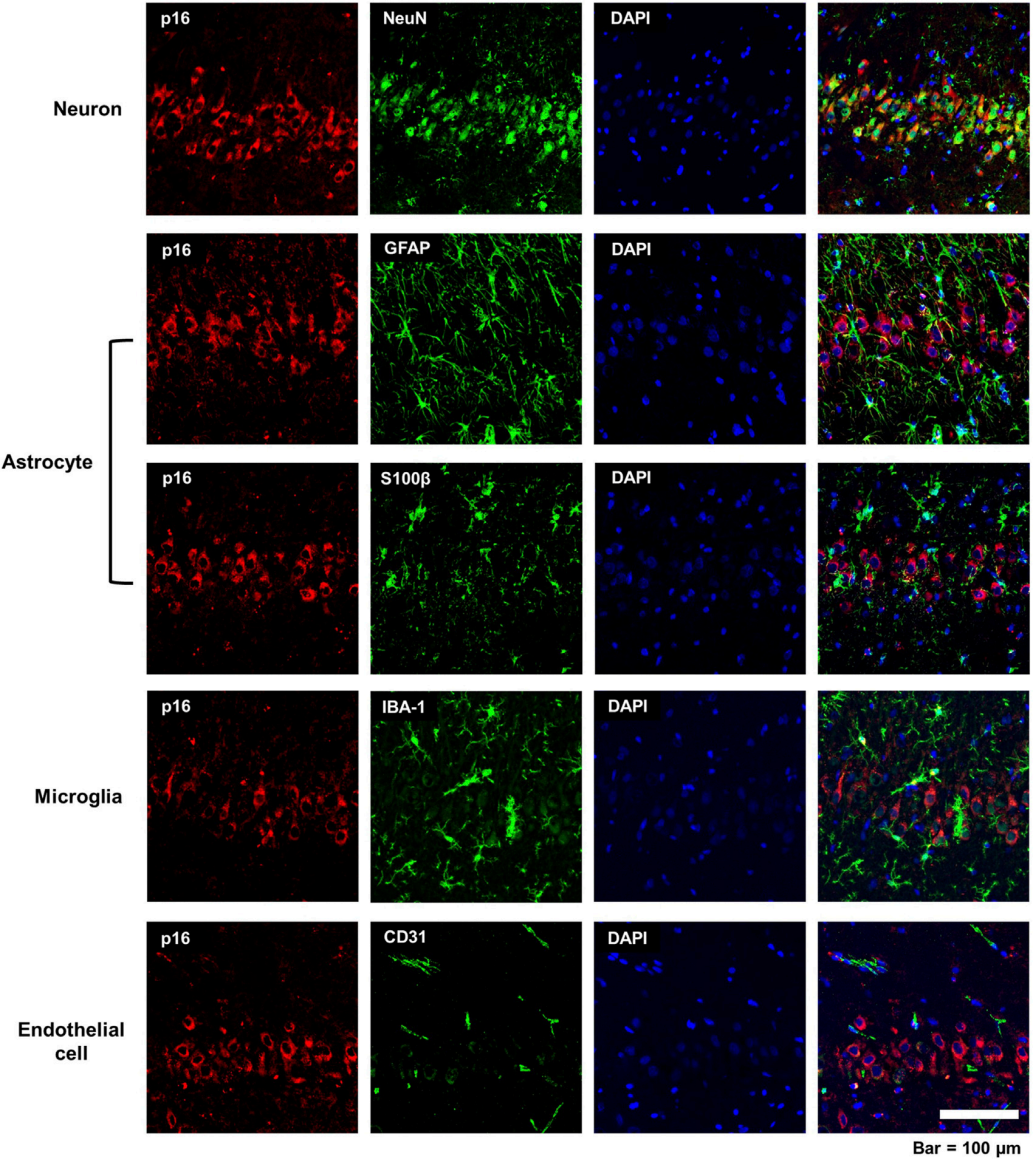
Cell specificity of p16 immunoreactivity in the acute DFP rat model. Representative photomicrographs of the CA3 of the hippocampus at 3-months post-DFP intoxication immunostained for p16 (red), a biomarker of senescence, and one of the following cell-specific biomarkers (green): NeuN for neurons; GFAP, astrocytes; S100β, astrocytes; IBA-1, microglia; or CD31, endothelial cell. Sections were counterstained with DAPI (blue) to identify cell nuclei. Scale bar = 100 μm.

To identify if p16 immunoreactivity was limited to specific cell types in the DFP brain at 3-months post-exposure, brain sections were co-labeled for p16 and cell type-specific markers, including NeuN for neurons (Mullen et al., 1992), GFAP and S100β for astrocytes (Eng and Ghirnikar, 1994; Raponi et al., 2007), IBA-1 for microglia/monocytes (Ito et al., 1998), and CD31 for endothelial cells (Mantovani and Dejana, 1998). As shown in representative photomicrographs of the CA3 region of the hippocampus of DFP-exposed animals at 3-months post-intoxication (Figure 3), p16 immuno-reactivity colocalized with NeuN immunoreactivity but not with the other cell type-specific biomarkers, suggesting that acute DFP intoxication specifically induced cellular senescence in neurons.

### 3.3 Cellular senescence persisted at 6-months post-DFP intoxication

**FIGURE 4:**
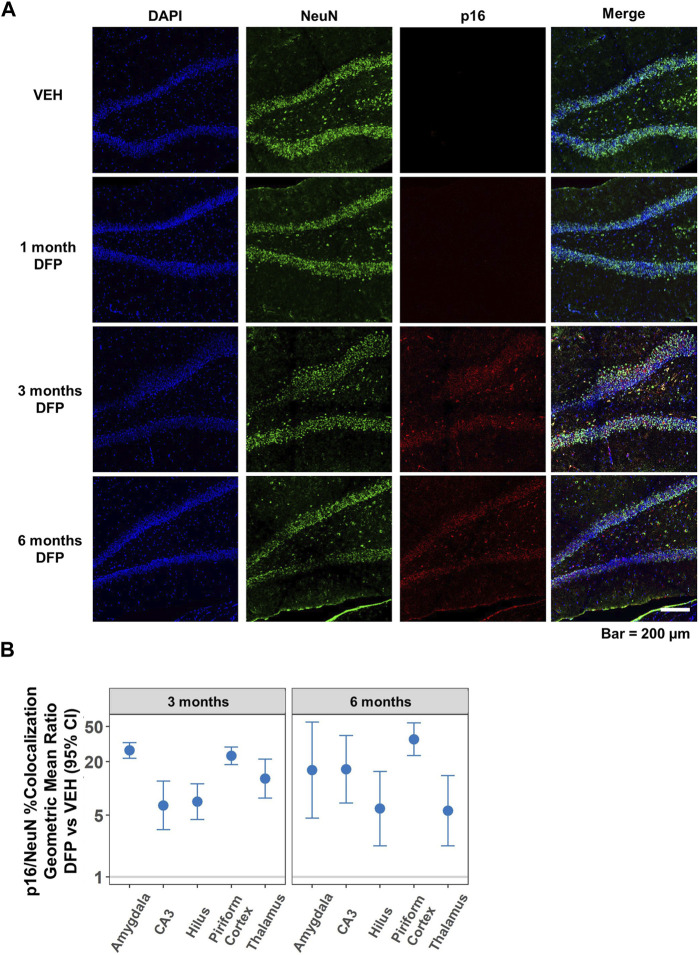
Temporal profile of p16 expression following acute DFP intoxication. **(A)** Representative photomicrographs of NeuN (green) and p16 (red) immunoreactivity in the dentate gyrus of the hippocampus at 1, 3, and 6 months post-DFP intoxication in comparison to VEH. Scale bar = 200 µm. **(B)** No p16 immunoreactivity was detected at 1-month post-exposure in any of the brain regions examined. However, at 3- and 6-months post-exposure, neuronal p16 expression was significantly increased in all brain region examined in the DFP vs. VEH animals. Dots represent point estimates of the geometric mean ratios (GMRs) of % NeuN-immunopositive cells co-labeled with p16 in DFP vs. VEH; bars represent the 95% confidence intervals. When the confidence interval includes 1, there is no statistical evidence of a significant difference between the two groups. All significant results (colored blue) remained significant after correction for false discovery rate (FDR).

The time course and persistence of cellular senescence in the brain following DFP-induced SE was examined by comparing p16 expression of DFP-intoxicated animals *versus* VEH controls at 1, 3, and 6 months post-exposure. As illustrated in representative photomicrographs of the dentate gyrus of the hippocampus (Figure 4A), relative to region-matched samples from VEH controls, DFP-intoxicated animals had significantly increased p16 immunoreactivity at 3- and 6-months post-exposure, but not at 1-month pose-exposure. Consistent with data shown in Figure 3, p16 immunoreactivity co-localized with NeuN immunoreactivity, and quantification of neuronal senescence confirmed significantly increased neuronal expression of p16 in the hippocampus (CA3 and dentate gyrus), amygdala, piriform cortex, and thalamus of DFP animals when compared to VEH controls at both 3- and 6-months post-exposure (Figure 4B; *p* < 0.001, GMR >5.6; the data presented as dot plots is provided in the Supplementary Material, Supplementary Figure S2). The difference between DFP and VEH animals in the percentage of NeuN immunopositive cells co-labeled for p16 varied by brain region and time point (*p* = 0.002), suggesting regional differences in the temporal profile of neuronal senescence in DFP animals.

Within the DFP group, p16 expression in the CA3 region of the hippocampus at 6-months post-exposure was two-fold higher than at 3-months post-exposure (GMR = 2.1%, 95% CI = 1.3–3.3, *p* = 0.002). In contrast, p16 expression at 6-months post-exposure was lower than at 3-months post-exposure in the hilus of dentate gyrus (GMR = 0.4%, 95% CI = 0.2–0.7, *p* = 0.002) and thalamus (GMR = 0.6%, 95% CI = 0.4–0.9, *p* = 0.01). There were no significant differences in p16 expression between 6 months and 3 months in any of the brain regions in VEH animals (*p* > 0.07).

### 3.4 Senescence-associated β-galactosidase (SA β-gal) coincides with p16 expression

**FIGURE 5:**
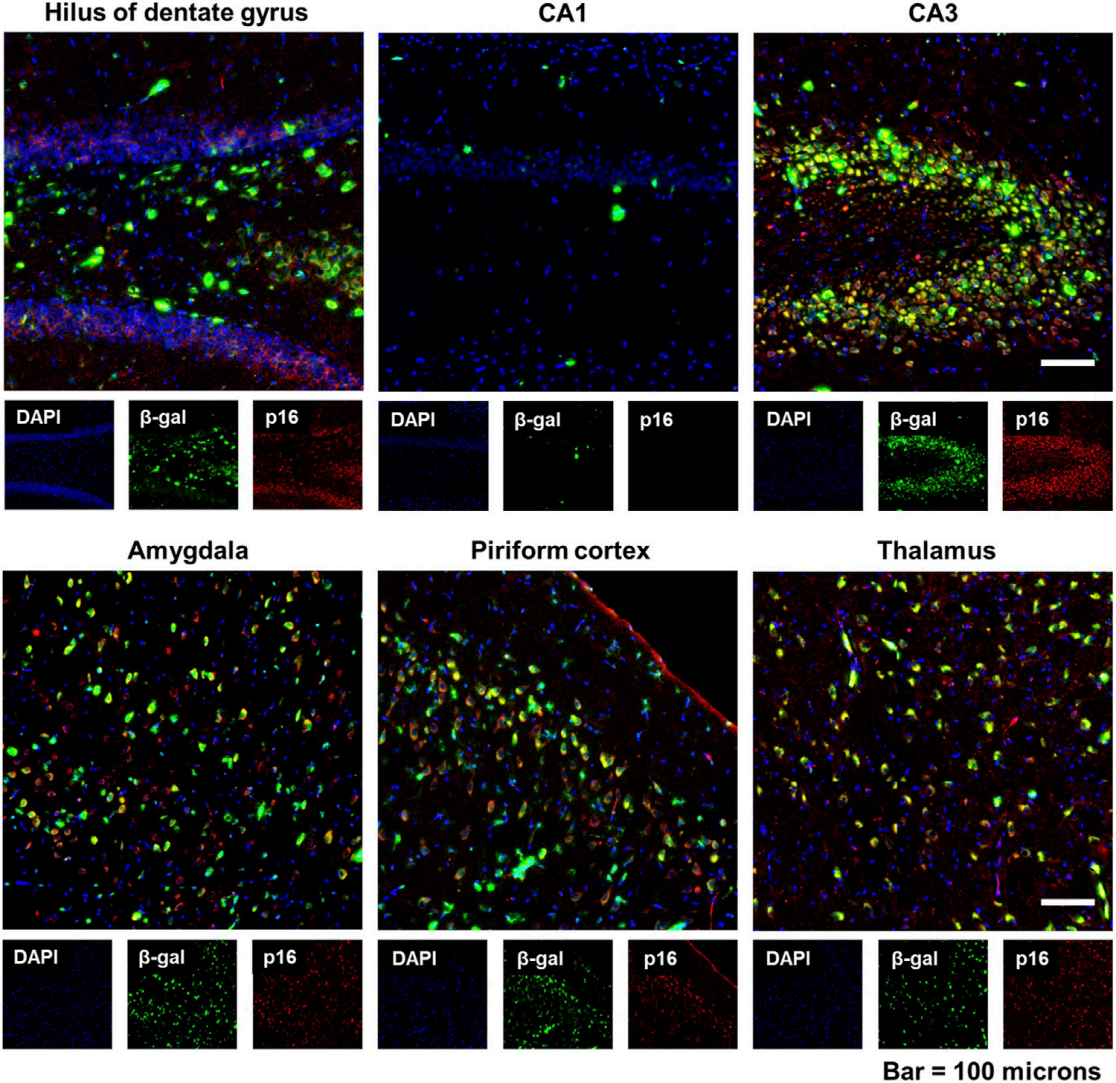
Expression of senescence-associated β-galactosidase (SA β-gal) activity coincided with increased p16 expression in the brain following acute DFP intoxication. Representative photomicrographs of fluorescent β-gal (green) and p16 (red) dual staining of the hippocampus (hilus of dentate gyrus, CA1, and CA3), amygdala, piriform cortex, and thalamus at 6 months post-DFP intoxication. Scale bar = 100 µm.

Senescence-associated β-galactosidase (SA β-gal) activity, which is a measure of lysosomal galactosidase activity at pH 6.0, is widely used as a biomarker of senescent cells both *in vitro* and *in vivo* (Lee et al., 2006). To corroborate our findings with p16 immunoreactivity, we stained brain sections from DFP animals for SA β-gal at 6-months post-exposure. As shown in the representative photomicrographs in Figure 5, fluorescent β-gal was detected in multiple brain regions of DFP animals and it coincided spatially with p16 expression.

### 3.5 Neuronal senescence overlaps with neurodegeneration in the DFP rat brain

We have previously reported that acute DFP intoxication increases neurodegeneration, evident as increased FluoroJade-C (FJC) staining, in multiple brain regions as early as 4 h post-exposure (Li et al., 2011) that persists at 6-months post-exposure (Supasai et al., 2020). To confirm these earlier observations in the current study, we used FJC staining on brain sections from DFP and VEH animals to visualize degenerating neurons (Schmued et al., 2005) in the amygdala, hippocampus, piriform and somatosensory cortices, and thalamus at 3- and 6-months post-DFP intoxication. Consistent with our previous report (Supasai et al., 2020), negligible FJC labeling was observed in brain sections of VEH animals, while in brain sections of DFP animals, FJC-labeled cells were observed in multiple brain regions, including the hippocampus (Supplementary Material, Supplementary Figure S1) at both 3- and 6-months post-intoxication. Quantitative analysis of the number of FJC-positive cells per unit area as a function of time post-exposure confirmed that acute DFP intoxication resulted in significant neurodegeneration (Supplementary Material, Supplementary Figure S1). The difference in FJC-labeled cells between DFP and VEH did not significantly differ by brain region or time, so an overall estimate of the group difference was obtained. The number of FJC-positive cells was significantly higher in DFP animals than VEH animals (GMR = 36.6%, 95% CI = 22.7–59.0; *p* < 0.001).

**FIGURE 6:**
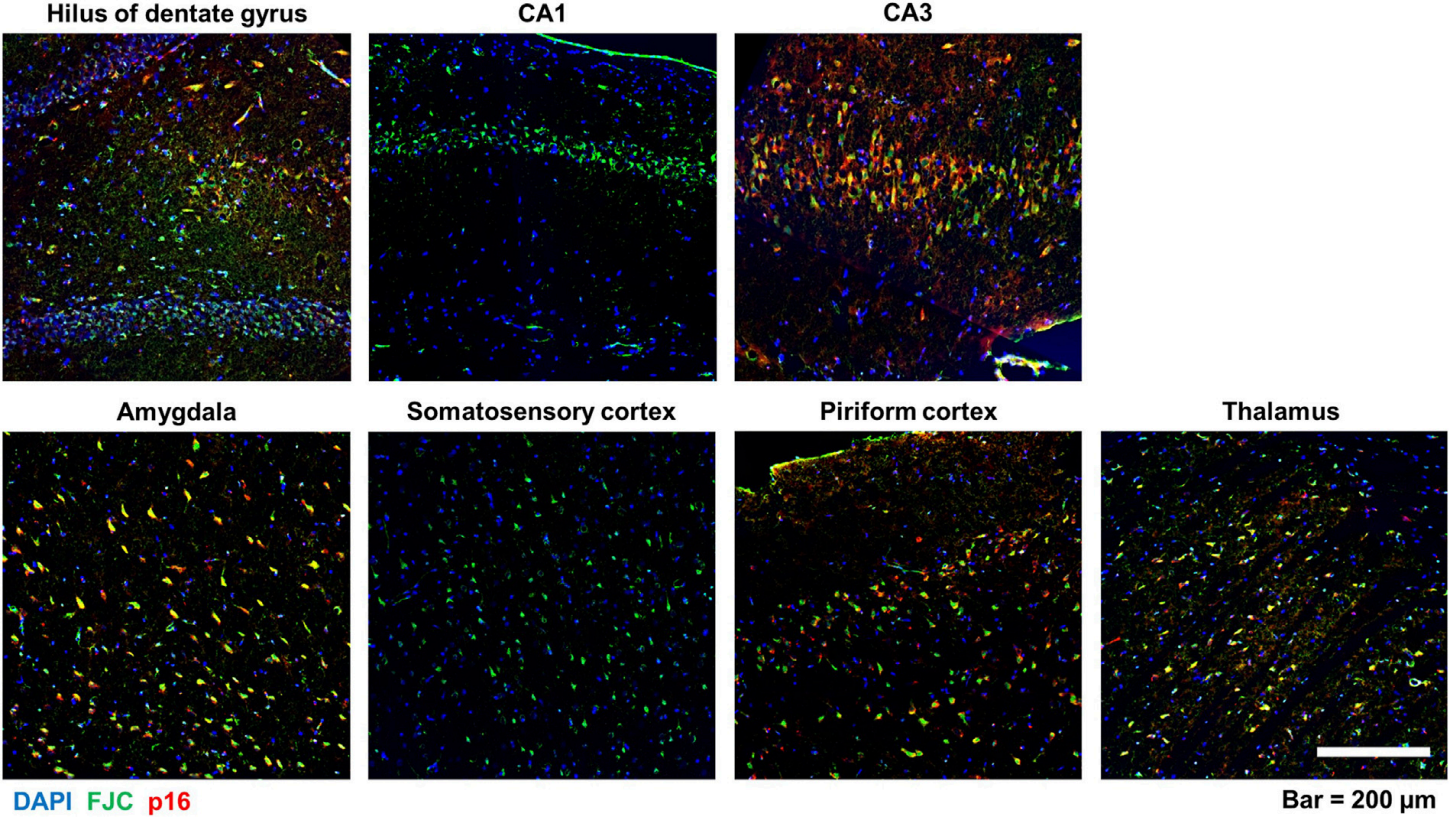
Colocalization of neuronal p16 expression and FJC-labeled cells in various brain regions following acute DFP intoxication. Representative photomicrographs of p16 immunoreactivity (red) and FJC staining (green) in multiple brain regions at 6-months post-DFP intoxication. Sections were counterstained with DAPI (blue) to identify cell nuclei. Scale bar = 200 µm.

**FIGURE 7:**
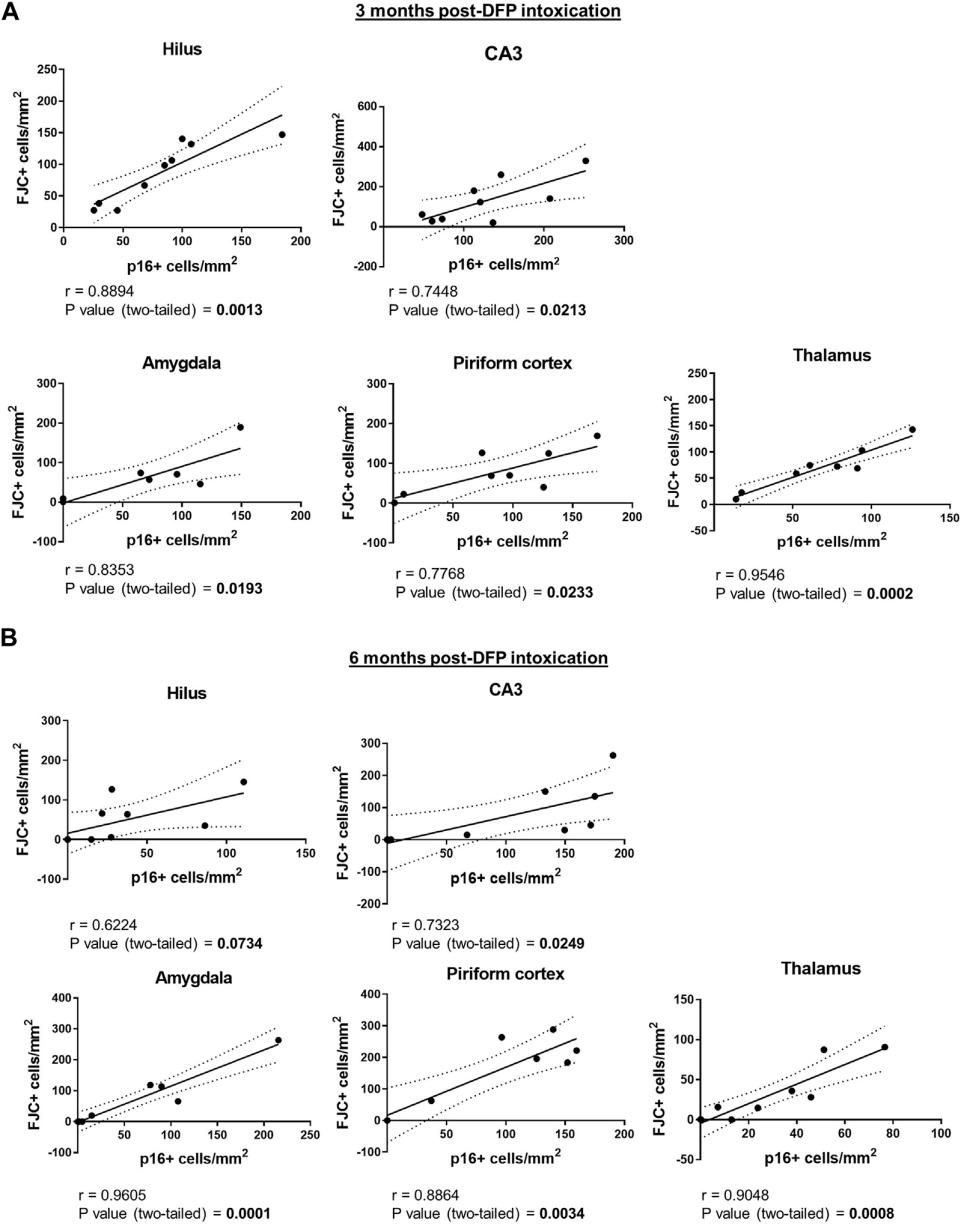
Neuronal senescence and neurodegeneration are positively correlated in multiple brain regions of DFP-intoxicated animals. **(A) (B)** Pearson correlation of p16-immunopositive *versus* FJC-labeled cells in the hilus, the CA3 of the hippocampus, amygdala, piriform cortex, and the thalamus at 3 **(A)** and 6 **(B)** months post-DFP intoxication.

To determine whether the spatiotemporal profile of neurodegeneration coincided with that of neuronal senescence, brain sections were immunostained for p16 and then labeled with FJC. There was considerable overlap of p16 immunoreactivity with FJC labeling, as shown in the representative photomicrographs of the hilus of the dentate gyrus, CA3 of the hippocampus, and the thalamus of DFP animals (Figure 6). Pearson correlational analysis of all animals from the 3-month cohort demonstrated a positive correlation between the presence of FJC-positive cells and p16-immunopositive cells in the hilus (r = 0.8894, *p* = 0.0013), CA3 (r = 0.7448, *p* = 0.0213), amygdala (r = 0.8353, *p* = 0.0193), piriform cortex (r = 0.7768, *p* = 0.0233), and thalamus (r = 0.9546, *p* = 0.0002) (Figure 7A). At 6-months post-exposure (Figure 7B), a positive correlation was found for CA3 (r = 0.7323, *p* = 0.0249), amygdala (r = 0.9605, *p* = 0.0001), piriform cortex (r = 0.8864, *p* = 0.0034), and thalamus (r = 0.9048, *p* = 0.0008), but not the hilus (*p* = 0.0734).

## 4 Discussion

There is increasing interest in the contribution of cellular senescence in the brain to the pathophysiology of brain aging and neurodegenerative disease (reviewed in Baker and Petersen, 2018, reviewed in Kritsilis et al., 2018, reviewed in Wissler Gerdes et al., 2020). In various animal models, the clearance of senescent cells via pharmacological approaches significantly improved the performance of animals in behavioral tests of cognitive function (Bussian et al., 2018; Zhang et al., 2019; Ogrodnik et al., 2021), providing proof-of-concept evidence linking cellular senescence to the cognitive deficits associated with aging- and neurodegenerative disease. Cognitive deficits are also associated with acute OP intoxication, and in the rat model of acute DFP intoxication, impaired learning and memory manifests during the weeks to months following DFP exposure (Brewer et al., 2013; Flannery et al., 2016; Rojas et al., 2016; Guignet et al., 2020). However, cellular senescence has not been investigated in the context of acute OP intoxication.

To test the hypothesis that acute OP intoxication promotes cellular senescence, we quantified the expression of well-characterized biomarkers of cellular senescence in the adult rat model of acute DFP intoxication. The hypothesis is supported by our findings. Specifically, we observed: 1) acute DFP intoxication significantly increased expression of p16 and SA β-gal in overlapping areas; 2) p16 immunoreactivity colocalized with a neuron-specific biomarker but not with biomarkers for astrocytes, microglia, or endothelial cells; and 3) neuronal senescence in the brain of DFP animals is spatially heterogeneous, with significant p16 immunoreactivity detected in the CA3 and hilus of the hippocampus, amygdala, piriform cortex, and thalamus, but not in the CA1 of the hippocampus or somatosensory cortex. In contrast to other well-documented neuropathologic consequences of acute OP intoxication, such as neuroinflammation, oxidative stress, and neurodegeneration which are significantly elevated during the first few days to weeks post-exposure (Li et al., 2011; Rojas et al., 2015; Flannery et al., 2016; Siso et al., 2017; Liang et al., 2018), p16 expression was not observed during the first month after DFP intoxication. Significant p16 expression was evident at 3-months post-DFP and remained at high levels through 6 months post-intoxication.

Various cell types in the brain have been reported to express features of cellular senescence in aging and neurodegenerative diseases including neurons (Jurk et al., 2012; Musi et al., 2018; Moreno-Blas et al., 2019; Vazquez-Villaseñor et al., 2020), astrocytes (Bhat et al., 2012; Chinta et al., 2018; Vazquez-Villaseñor et al., 2020), microglia (Bussian et al., 2018) and oligodendrocyte progenitor cells (Zhang et al., 2019). Our observation of delayed neuronal senescence in the brain of the DFP rat months post-exposure adds to the nascent body of literature identifying senescent phenotypes in postmitotic cells. This finding calls into question the widely held view that cellular senescence is a property of proliferating cells (reviewed in Gorgoulis et al., 2019, reviewed in von Zglinicki et al., 2020).

It was first shown by Jurk and colleagues that DNA damage leads to a senescence-like state in mature postmitotic neurons *in vivo* via a p21-dependent mechanism (Jurk et al., 2012). Reports of altered expression of cellular senescence-related cell cycle markers in postmitotic neurons challenge the current dogma that neurons exit from the cell cycle once they are terminally differentiated (Copani et al., 2001; Wani et al., 2017; Moreno-Blas et al., 2019; Barrio-Alonso et al., 2020). It is worth noting that aberrant expression of cell cycle proteins in postmitotic cells often results in abnormal cell cycle reentry and subsequent cell death (reviewed in Xia et al., 2019). For example, differentiated PC12 cells exposed to the OP pesticide dichlorvos for 12 h exhibited increased expression of p53, cyclin-D1 and pRb, but decreased expression of p21(Wani et al., 2017). Further cell cycle analysis via flow cytometry confirmed that dichlorvos shifted the distribution of cells among the different phases of the cell cycle, with fewer cells in the G0/G1 phase and more in the S and G2/M phases compared to controls. These effects of dichlorvos coincided with increased expression of pro-apoptotic proteins Bax and cytochrome c and decreased expression of anti-apoptotic protein Bcl-2, suggesting that dichlorvos caused neuronal cell damage potentially via triggering re-entry of differentiated PC12 cells into the cell cycle, which led to apoptotic cell death.

p16 is a cyclin-dependent kinase inhibitor that prevents cell cycle progression (Sherr and Roberts, 1999). Thus, it is possible that in the acutely intoxicated DFP rat, increased expression of p16 might be potentially beneficial initially by allowing stressed neurons to enter a senescent state to avoid cell cycle re-entry and subsequent apoptosis. However, accumulation of senescent neurons in the brain may eventually result in neural network dysregulation and chronic inflammation. In support of this possibility, senescent neurons have been shown to adopt the senescence-associated secretory phenotype (SASP) and become pro-inflammatory (reviewed in Chinta et al., 2015; Jurk et al., 2012, reviewed in Sah et al., 2021). While we did not measure the SASP in this study, we propose the testable hypothesis that senescent neurons in the DFP brain contribute to persistent DFP-induced neuroinflammation (Supasai et al., 2020) by secreting SASP factors, such as pro-inflammatory cytokines and exosomes, to induce senescence in neighboring neurons in a paracrine fashion as previously demonstrated in other models (Nelson et al., 2012).

In this study, we observed a positive correlation between senescent and degenerating neurons in the amygdala, hippocampus, piriform cortex, and thalamus, as indicated by p16 immunoreactivity and FJC labeling. This raises questions as to whether cellular senescence drives neurodegeneration or, conversely, whether neurodegeneration promotes neuronal senescence. However, our observation that not all brain regions previously reported to exhibit significant FJC labeling at 3- and 6-months post-DFP intoxication (Supasai et al., 2020) exhibited p16 immunoreactivity suggests that neuronal senescence and neurodegeneration are likely independent but parallel processes that occur months after acute DFP intoxication.

A key question raised by our study concerns the mechanism(s) by which acute DFP intoxication causes delayed neuronal senescence. Previous studies have demonstrated that intoxication with a single seizurogenic dose of DFP triggers a robust neuroinflammatory response (Liu et al., 2012; Rojas et al., 2015; Flannery et al., 2016; Siso et al., 2017; Guignet et al., 2020) and upregulates multiple biomarkers of oxidative stress (Zaja-Milatovic et al., 2009; Liang et al., 2018; Putra et al., 2020a; Putra et al., 2020b; Guignet et al., 2020) within hours that persist for weeks to months post-exposure. Cellular senescence can be induced by neuroinflammation and oxidative stress (reviewed in Gorgoulis et al., 2019, reviewed in Martínez-Zamudio et al., 2017, reviewed in Baker and Petersen, 2018, reviewed in Walton and Andersen, 2019), suggesting potential mechanisms underlying neuronal senescence in the brain of DFP rats. Further studies will be required to assess causal relationships between neuroinflammation, oxidative stress, and neuronal senescence following acute OP intoxication and evaluate their contributions to adverse neurological outcomes.

While we observed delayed but persistent neuronal senescence in brain regions that are critically involved in cognitive behavior after acute DFP intoxication, there are several limitations of this study that need to be addressed. First, these findings need to be corroborated by characterizing expression of additional biomarkers of cellular senescence, such as p21, lamin B1, and SASP. Second, it would be of value to determine which neuronal cell type(s) are expressing p16 and whether treatments targeting cellular senescence mitigate not only neuronal senescence, but also cognitive impairment. Third, further studies are warranted to determine whether cellular senescence is a phenotype generalized across OPs.

In summary, we present novel data demonstrating that acute DFP intoxication caused delayed cellular senescence, specifically in neurons. Spatiotemporal characterization of neuronal p16 expression indicated that senescence developed between 1- and 3-months post-DFP exposure and persisted at 6-months post-exposure. In addition, we identified a positive correlation between neurodegeneration and neuronal senescence in the amygdala, hippocampus, piriform cortex, and thalamus; however, it is not clear whether there is a causal relationship between these two pathogenic outcomes because neurodegeneration was observed in brain regions at 6 months post-DFP intoxication that exhibited negligible p16 immunoreactivity. These findings implicate cellular senescence as a potential pathogenic mechanism linking acute OP intoxication to chronic neurotoxic outcomes, identifying cellular senescence as a potential therapeutic target for protecting brain function in individuals acutely intoxicated with OPs.

## Data Availability

The original contributions presented in the study are included in the article/Supplementary Material, further inquiries can be directed to the corresponding author.
