# Supplementary material for: Acute intoxication with diisopropylfluorophosphate promotes cellular senescence in the adult male rat brain
Source: Front Toxicol. 2024 Apr 30;6:1360359. doi: 10.3389/ftox.2024.1360359 (PMC11091247; doi:10.3389/ftox.2024.1360359)
Supplement: Supplementary file 1 [file DataSheet1.DOCX]

Supplementary Material for Tsai et al.

Supplemental methods: Custom module used to quantify p16/NeuN colocalization.

**
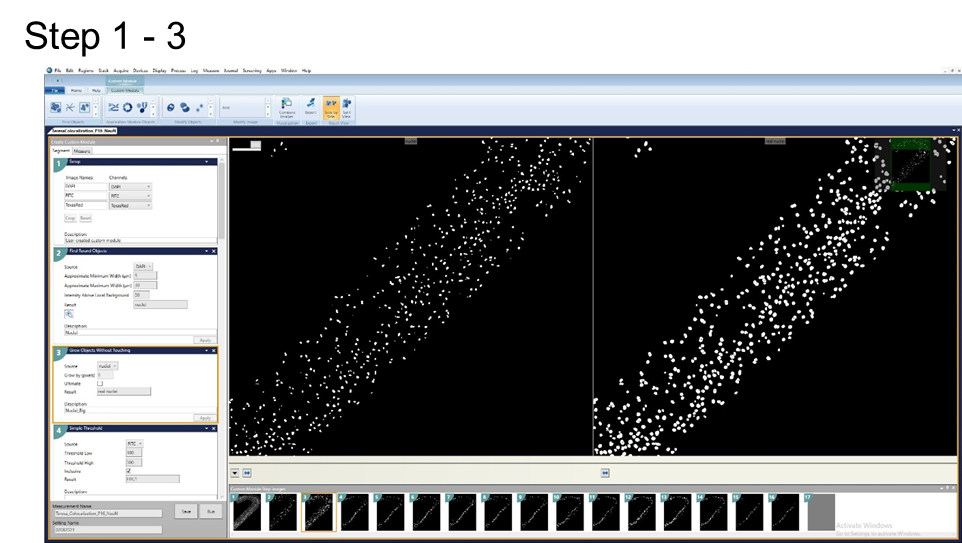
**

**
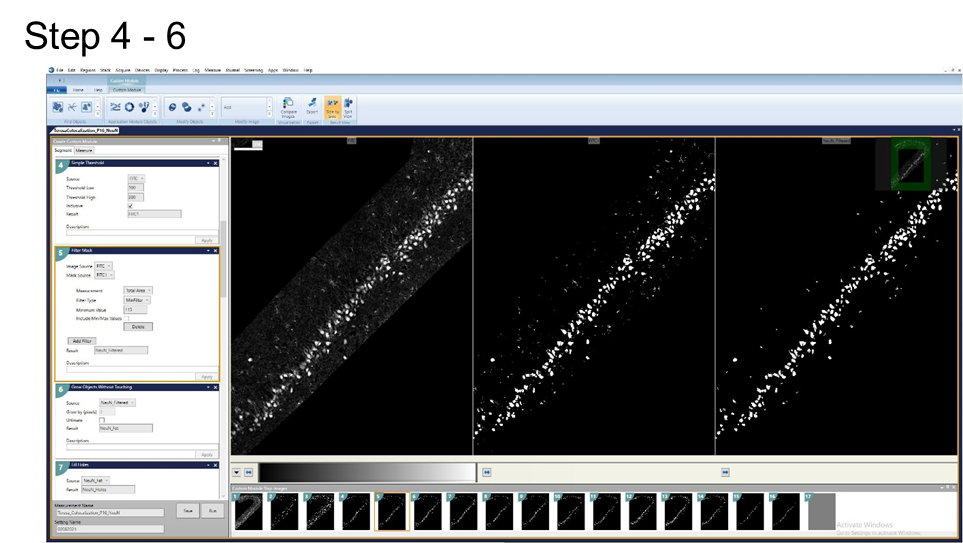
**

**
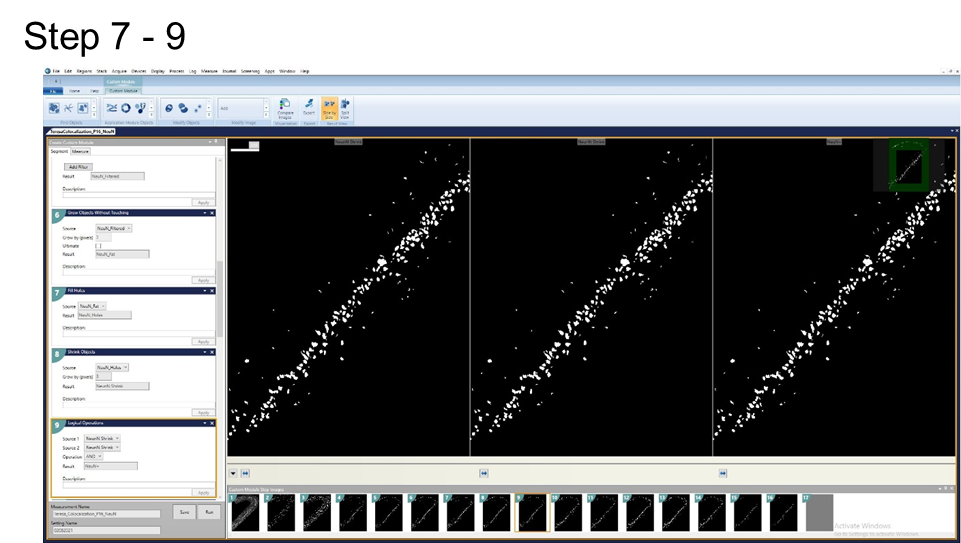
**

**
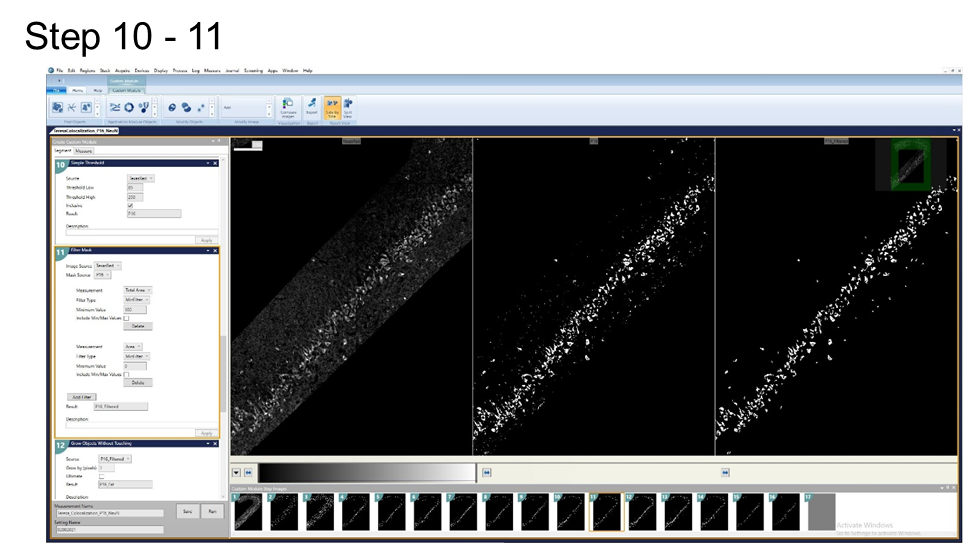
**

**
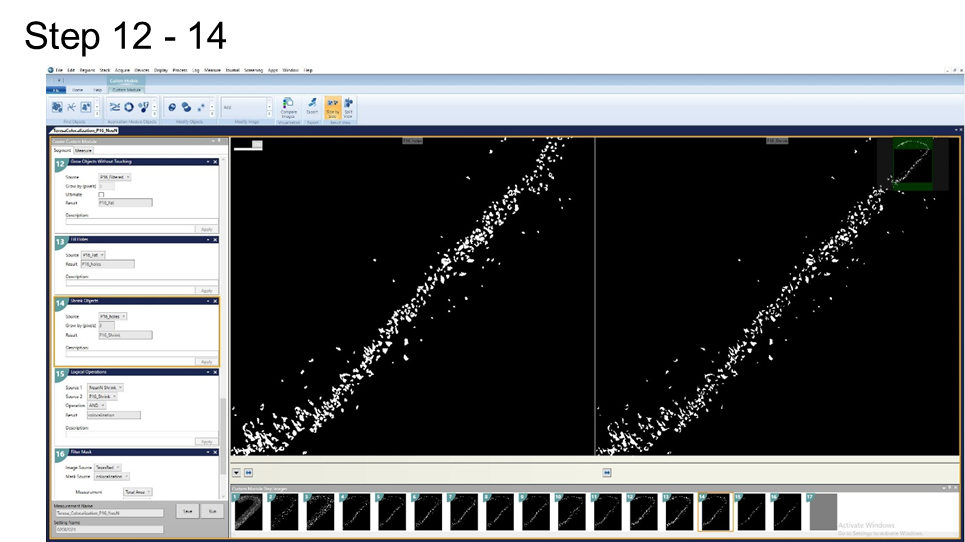
**

**
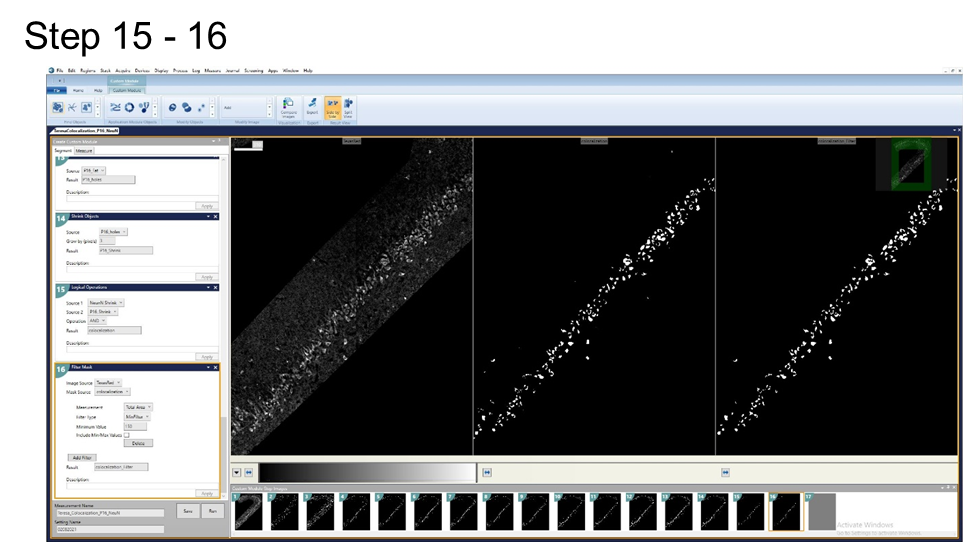
**

**Supplemental method. Steps used to measure p16/NeuN colocalzation using Custom Module Editor from Molecular Devices.** Below are the steps included for building the analysis: 1) Setup with DAPI, FITC, and TexasRed wavelengths; 2) Find Round Objects: Identify nuclei on the DAPI image; 3) Grow Objects Without Touching: Enlarge the nuclei by 8 pixels; 4) Simple Threshold for FITC (set low at 100 and high at 300): Enable and adjust the threshold of the image manually to help segment the features from the background on the FITC image; 5) Filter Mask: Remove objects from the mask based on measurement values of the objects by using Total Area and MinFilter value at 115; 6) Grow Objects Without Touching: Enlarge the identified objects on the FITC image by 3 pixels (“NeuN_Fat”); 7) Fill Holes: Fills holes in objects identified by Step 6, which allows the filled area to be included in measurement data (“NeuN_Holes”); 8) Shrink Objects: Shrink objects from “NeuN_Holes” by 3 pixels (“NeuN_Shrink”); 9) Logical Operations: Have the results for NeuN+ objects; 10) Simple Threshold for TexasRed (set low at 85 and high at 250): Enable and adjust the threshold of the image manually to help segment the features from the background on the TexasRed image (“P16”); 11) Filter Mask: Remove objects from the mask based on measurement values of the objects by using Total Area and MinFilter value at 100; 12) Grow Objects Without Touching: Enlarge the identified objects on the Texas Red image by 3 pixels (“P16_Fat”); 13) Fill Holes: Fills holes in objects identified by Step 12, which allows the filled area to be included in measurement data (“P16_holes”); 14) Shrink Objects: Shrink objects from “P16_holes” by 3 pixels (“P16_Shrink”); 15) Logical Operation: Have the colocalization results for NeuN+ objects with P16 objects; 16) Filter Mask: Remove objects from the mask based on the measurement from Step 15 by using Total Area and MinFilter value at 150 (“colocalization_Filter”)


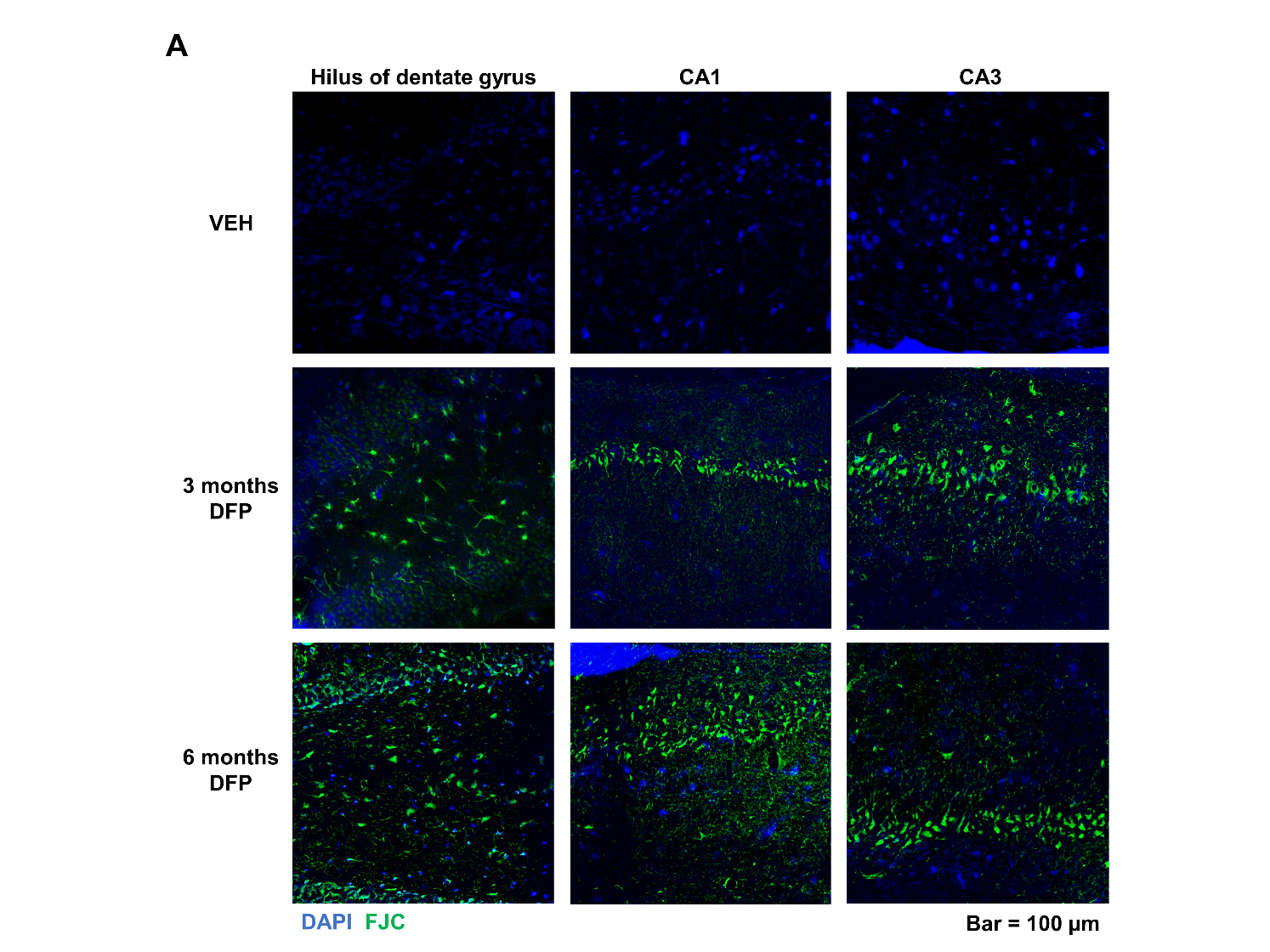


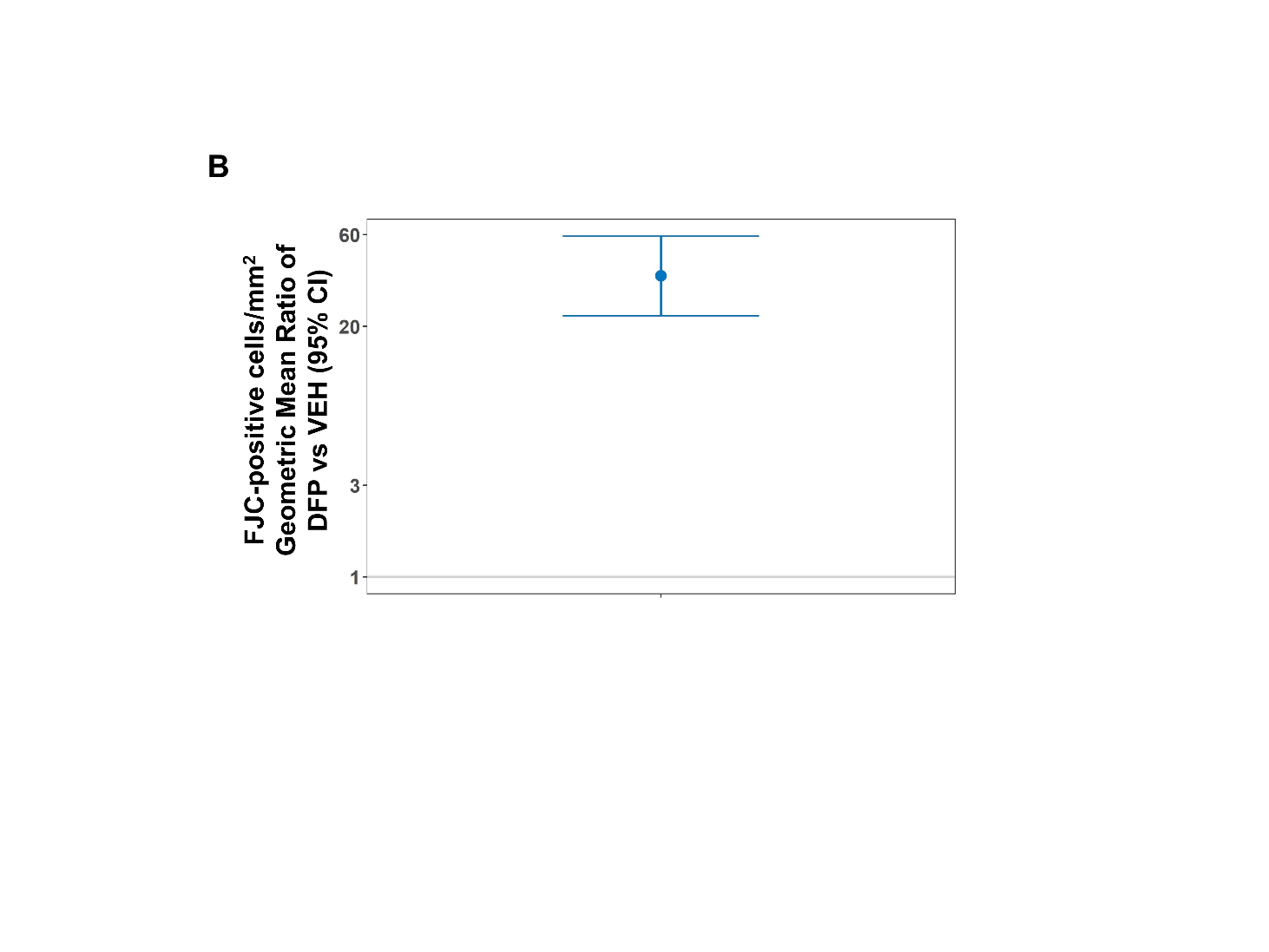


**Supplementary Figure S1. DFP caused neurodegeneration that persisted at 3- and 6-months post-exposure. (A)** Representative photomicrographs of FJC labeling (green) in the hippocampus (hilus of dentate gyrus, CA1 and CA3) of rats at 3 and 6 months following exposure to vehicle (VEH) or DFP. All sections were counterstained with DAPI (blue) to identify cell nuclei. Scale bar = 100 µm. **(B)** Analysis of FJC-labeled cells per unit area. Dots represent point estimates of the geometric mean ratios (GMRs) of FJC-positive cells per mm^2^ in DFP vs. VEH; bars represent the 95% confidence intervals. When the confidence interval includes 1, there is no statistical evidence of a significant difference between the two groups. Significant results are colored in blue. No statistically significant differences between brain region and time post-exposure were found, so all brain regions and time points were collapsed.


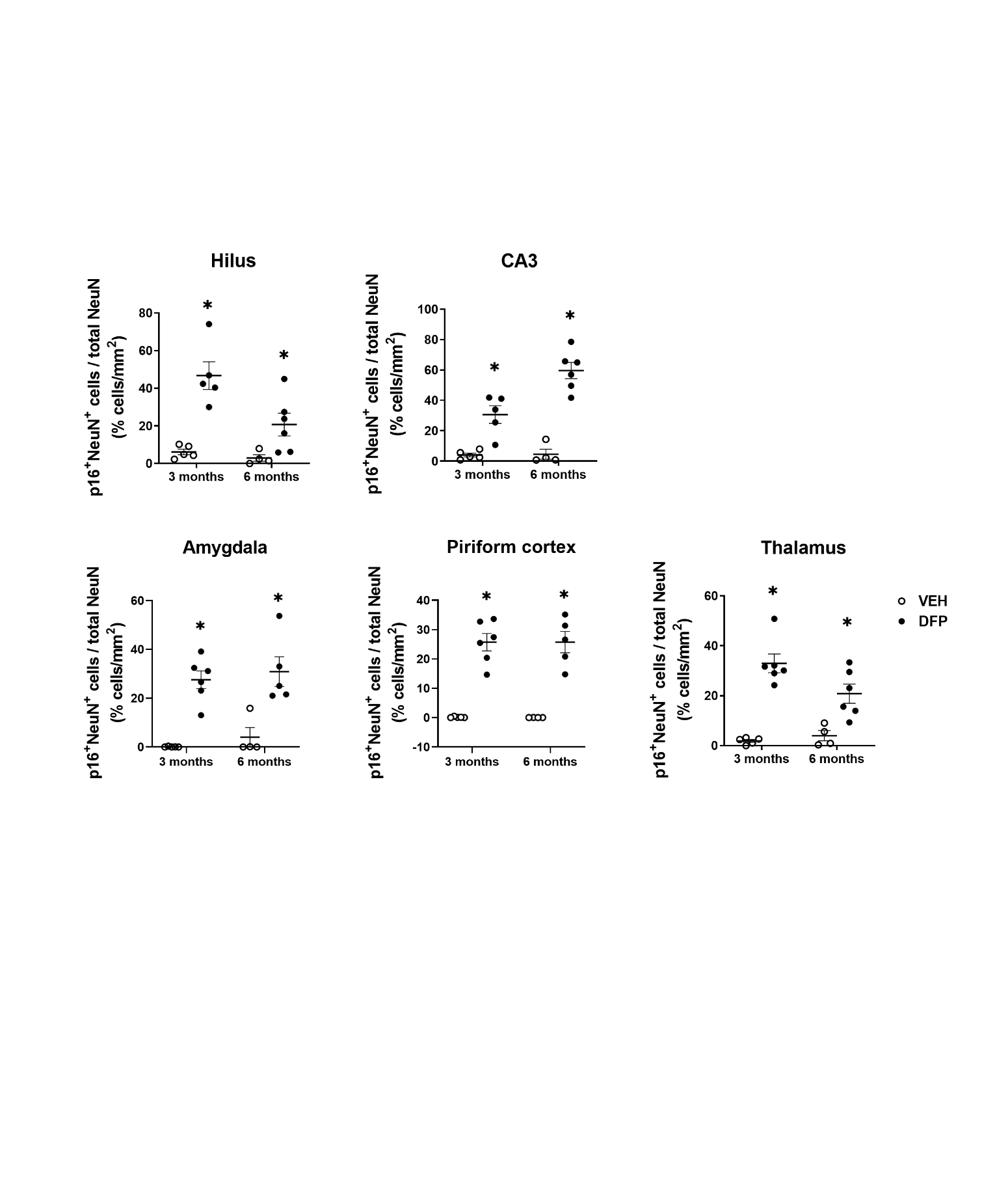


**Supplementary Figure S2. Acute DFP intoxication increases senescent neurons in several brain regions at 3 and 6 months post-exposure**. Analysis of percentage of NeuN-immunopositive cells that co-express p16 immunoreactivity. Horizontal lines in dot plots represent the mean (n = 4 - 5 for VEH groups; n = 5 - 6 for DFP groups); whickers: the SEM; and dots: data from individual animals. *Significantly different (p < 0.05) from the VEH group as determined by two-way ANOVA with groups and time points as variables and post-hoc Tukey test.
